# Predictive Value of Preoperative Neutrophil-Lymphocyte and Platelet-Lymphocyte Ratio in Determining the Stage of Colon Tumors

**DOI:** 10.7759/cureus.18381

**Published:** 2021-09-29

**Authors:** Veysel Barış Turhan, Abdulkadir Ünsal, Halil Fatih Gök, Bülent Öztürk, Doğan Öztürk, Gulcin Guler Simsek, Hakan Buluş

**Affiliations:** 1 General Surgery, Health Sciences University Keçiören Training and Research Hospital, Ankara, TUR; 2 Pathology, University of Medical Sciences, Gülhane Training and Research Hospital, Ankara, TUR

**Keywords:** tnm, serum biomarkers, stage, neutrophil-leukocyte ratios, platalet-lymphocyte ratios

## Abstract

Introduction

Biomarkers such as the neutrophil-lymphocyte ratio (NLR) and the platelet-lymphocyte ratio (PLR) are associated with the colon tumor stage and prognosis. Therefore, in our study, we investigated whether these biomarkers are important in determining the colon cancer stage.

Materials and methods

The outcomes in 268 patients operated on with the diagnosis of colon cancer between January 2011 and March 2019 were retrospectively analyzed. The relationship of the stage of the patients with the NLR or PLR was evaluated. In addition, according to the stage of colorectal tumors, stage I and other stages (stages II, III, and IV) were compared in terms of NLR and PLR. Groups that had lymph node (LN) metastasis were compared with those that did not. Finally, groups with and without metastasis were also compared.

Results

In our cohort, 144 patients (57.6%) were male, and 84 (42.4%) were female. The mean age was found to be 68.28 ±12.71 years. The patients were evaluated according to their stages: 26 patients were stage I, 78 patients were stage II, 75 patients were stage III, and 19 patients were stage IV. There was a significant difference in NLR values between the groups (p: 0.05). Also, 104 patients were LN-negative (stages I-II), and 94 patients were LN-positive (stages III-IV). When PLR was compared between the two groups, no significant difference was found between tumor stages and these values (p: 0.099). However, there was a significant difference in NLR values (p: 0.034).

Conclusion

Based on our findings, it has been concluded that increased PLR may not be associated with the colon cancer stage. However, the increase in NLR was found to be correlated with tumor stage and LN metastasis.

## Introduction

Colon cancer is the most common gastrointestinal system cancer, and colorectal cancer is the third most common cause of death from cancer in the United States [1]. About two-thirds of the disease is confined to the colon, and more than 106,000 new colon cancer cases are diagnosed each year [2]. Its frequency gradually increases after the age of 50 years and reaches its peak after the age of 80 years. While the average age of diagnosis in males is 63 ±13.65 years, it is 62 ±12.71 years in females [3].

Genetic and environmental factors are crucial in the etiology of the disease. It is known that genetic factors play an important role in the development of the disease [4]. Hereditary diseases are the leading causes. The presence of colorectal cancer in the family is another risk factor. With the presence of colorectal cancer in a first-degree relative, the risk increases by 1.7 times, while the risk increases by 2.7 times when more than two colorectal cancers are found, and by 5.3 times in the presence of colorectal cancer in relatives under 45 years of age [5].

Clinical stage, tumor histopathology, tumor localization, and lymph node (LN) numbers are widely used to predict colon cancer prognosis [6-8]. However, the majority of the population with the same clinical stage and tumor differentiation may have different prognoses. Therefore, additional prognostic indicators are needed to identify the high-risk subgroup and optimize colon cancer therapeutic strategies.

There is increasing evidence for the roles that local immune response and systemic inflammation play in the progression of tumors and survival of patients with cancer [9-11]. Based on this theory, parameters such as neutrophil-lymphocyte ratio (NLR) and platelet-lymphocyte ratio (PLR) are associated with the tumor stage, and therefore with the prognosis [12]. These biomarkers have attracted increasing attention as they can be obtained easily and inexpensively via preoperative blood tests. The NLR is calculated by dividing the peripheral neutrophil count by the lymphocyte count. PLR is calculated by dividing the number of peripheral platelets by the number of lymphocytes.

Several studies have shown that PLR is significantly associated with clinical outcomes in various malignancies such as thyroid cancer [13], breast cancer, and gastric cancer [13-15]. In contrast, some studies do not support this conclusion [16]. Many studies have investigated the predictive value of these biomarkers in predicting tumor stage; the results have been insignificant, but the number of patients studied was small [17]. Their association with tumor stage, presence of LN metastases, and relationship with metastasis have not been well evaluated in colon cancer patients.

In light of this, this study aimed to evaluate NL and PLR values in colon cancer and determine an accurate prognostic indicator for predicting clinical stages and optimizing therapeutic strategies.

## Materials and methods

After obtaining the ethics committee approval, the outcomes in 268 patients who were operated on with the diagnosis of colon cancer in the General Surgery Clinic between January 2011 and March 2019 were retrospectively analyzed. Patients with diseases that might affect leukocyte, platelet, and neutrophil counts, patients with an inflammatory condition, and patients whose data could not be fully accessed were excluded from the study. Demographic data, tumor localization, tumor stages, routine biochemical and hemogram parameters were recorded. NLR and PLR values were calculated for each patient based on the whole blood evaluation performed in the preoperative preparation stage. The pathology and tomography results of the patients were examined, and their stages were classified according to the TNM classification.

Further, the relationship between the stages of the patients and the NLR or PLR was evaluated. Also, stage I and other groups were evaluated separately. The group with colon cancer and LN metastasis in the pathology and the group without LN metastases were compared. In addition, groups with and without distant metastasis were compared.

Analyses were performed using SPSS Statistics 22.0 (IBM, Armonk, NY) for Windows statistical program. Descriptive statistics (i.e., mean, SD, median, minimum, and maximum) for numerical variables and frequency distributions for categorical variables were reported. As the numerical variables did not show normal distribution in terms of the relationship between colon cancer stages and NLR and PLR, the hypothesis tests were performed with nonparametric estimation methods. The Mann-Whitney U and Kruskal-Wallis tests were used to compare NLR and PLR with other categorical variables.

## Results

The outcomes in 268 patients who underwent colon resection were analyzed. Patients whose data could not be accessed, whose pathology result was not malignant, and those who did not meet the study criteria were excluded from the study. As a result, 198 patients were included in the final analysis. The patients' demographic data are shown in Table 1; 114 of the patients (57.6%) were male, and 84 (42.4%) were female. The mean age was 68.28 ±12.71 years. There was no statistically significant difference between women's and men's NLR/PLR values (p: 0.334, p: 0.932, respectively).

**Table 1:** Comparison of demographic characteristics and laboratory findings with stage I-II and stage III-IV tumors *Statistically significant NLR: neutrophil-to-lymphocyte ratio; PLR: platelet-to-lymphocyte ratio; SD: standard deviation

Variables	Total (n=198)	Stages I-II (n=104)	Stages III-IV (n=94)	P-value
	Mean ±SD	Mean ±SD	Mean ±SD	
Age, years	68.28 ±12.71	67.74 ±12.41	67,86 ±13.025	0.624
Platelet, K/mL	334.30 ±123.79	336.78 ±124.76	332.048 ±123.466	0.789
Neutrophil, K/mL	7.38 ±3.97	6.62 ±3.60	8.06 ±4.186	0.011*
Lymphocyte, K/ml	1.725 ±0.742	1.84 ±0.737	1.62 ±0.734	0.036*
NLR	5.56 ±5.31	4.548 ±4.84	6.49 ±5.57	0.034*
PLR	233.02 ±134.32	216.53 ±129.17	247.932 ±137.72	0.099

The tumor was in the rectum in 18 patients, in the rectosigmoid region in 87 patients, in the descending colon in 36 patients, in the transverse colon in 12 patients, and in the ascending colon in 45 patients. When compared in terms of localization, no significant difference was found between localization and NLR/PLR values (p: 0.139, p: 0.149) (Table 2).

**Table 2:** Tumor localization NLR: neutrophil-to-lymphocyte ratio; PLR: platelet-to-lymphocyte ratio

Tumor localization	N=198	NLR, mean ±SD	PLR, mean ±SD
Rectum	18	5.548 ±3.84	219.53 ±129.17
Rectosigmoid	87	6.148 ±3.76	247.932 ±137.72
Ascending colon	36	4.948 ±3.94	226.63 ±129.56
Transverse colon	12	4.548 ±4.64	247.932 ±137.56
Descending colon	45	4.648 ±4.75	221.57 ±130.15

When the pathology results were examined, 41 patients had poorly differentiated adenocarcinoma, 98 patients had moderately differentiated adenocarcinoma, 39 patients had well-differentiated adenocarcinoma, and 20 patients had other cancers. No significant difference was found between NLR and PLR values (p: 0.337, p: 0.285) (Table 3).

The patients were evaluated according to their stages: 26 patients were stage I, 78 patients were stage II, 75 patients were stage III, and 19 patients were stage IV. Groups did not show homogeneity according to NLR values. A significant difference was found in NLR values between groups in the Kruskal-Wallis test (p: 0.05). It was seen that this difference was due to stage I and stage III in Tamhane's post hoc analysis (p: 0.001) (Table 4).

**Table 3:** Histopathology NLR: neutrophil-to-lymphocyte ratio; PLR: platelet-to-lymphocyte ratio; SD: standard deviation

Tumor histopathology	N=198	NLR, mean ±SD	PLR, mean ±SD
Poorly differentiated adenocarcinoma	41	6.148 ±3.84	231.53 ±130.17
Moderately differentiated adenocarcinoma	96	5.948 ±3.76	247.132 ±138.81
Well-differentiated adenocarcinoma	39	4.842 ±4.84	231.63 ±124.76
Other types	20	4.748 ±3.64	251.932 ±137.26

**Table 4:** Tamhane's post hoc analysis *Values that are statistically significant are indicated in bold NLR: neutrophil-to-lymphocyte ratio

Tamhane's post hoc analysis		
Dependent variable: NLR		P-value
Stage I	Stage II	0.114
	Stage III	0.001*
	Stage IV	0.099
Stage II	Stage I	0.114
	Stage III	0.282
	Stage IV	0.773
Stage III	Stage I	0.001*
	Stage II	0.282
	Stage IV	0.325
Stage IV	Stage I	0.099
	Stage II	0.773
	Stage IV	0.802

**Figure 1:**
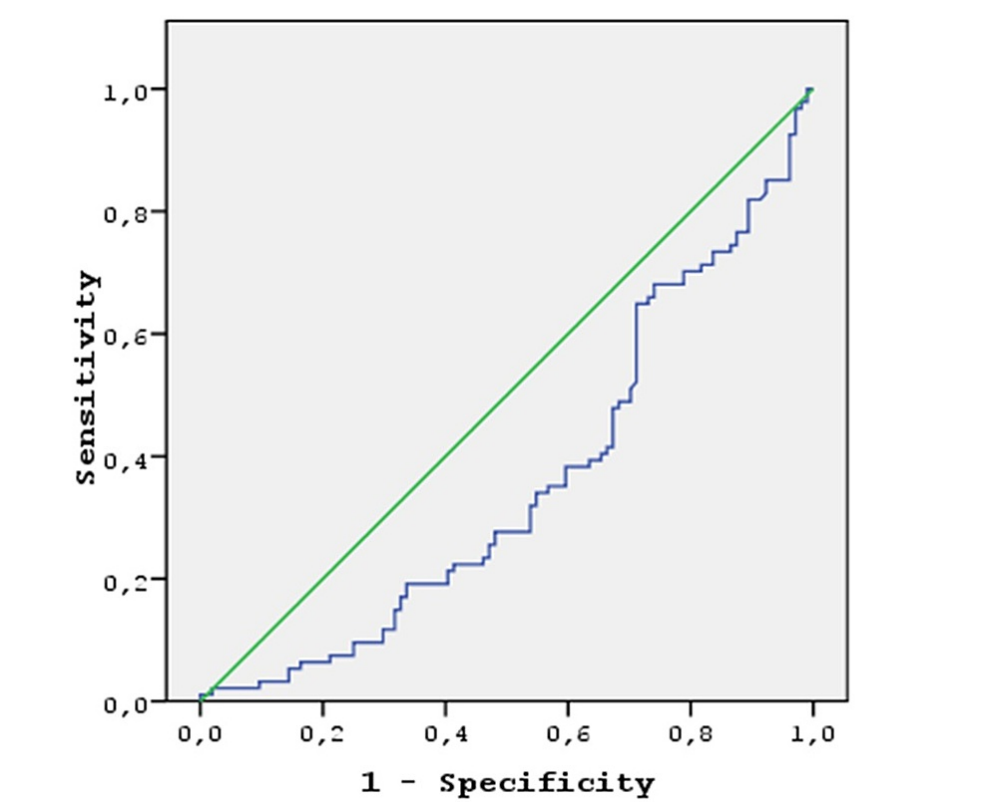
ROC curve for NLR *NLR ROC curve, cut-off=3.7008 sensitivity: 40.8%, specificity: 40.1%, AUC: 0.358 AUC: area under the curve; NLR: neutrophil-to-lymphocyte ratio; ROC: receiver operating characteristic

Also, 104 patients were LN-negative (stages I-II), and 94 patients were LN-positive (stages III-IV) (Table 1). When PLR was evaluated between the two groups, no significant difference was found between tumor stages and these values (p: 0.099). However, there was a significant difference in NLR values (p: 0.034). For NLR values, the area under the curve value was found to be 0.358. When the cut-off value was 3.7, the sensitivity was 40% and specificity 40%. Stages I, II, and III (group 1) and stage IV (group 2) were compared. The presence or absence of metastasis did not affect the NLR and PLR values in a statistically significant manner (p: 0.064). Figure 1 illustrates the ROC curve for NLR.

## Discussion

In this study, it was concluded that PLR is not effective in predicting colon cancer stages. In contrast, there was a significant relationship between NLR values and colon cancer stage. However, these results' sensitivity and specificity values that can be used in routine practice were low. Using the cut-off value we determined, predictions can be made at least before emergency surgeries using this easily applicable biomarker.

Similar to our study, Yıldırım et al. showed that PLR values were not effective in their study in which they investigated the effect of biomarkers in predicting staging in testicular tumors [17]. However, in this study, NLR values were also found to be insignificant. This may be attributed to the low number of patients. Despite the high number of cases in our study, the findings were not very significant, perhaps because they were insufficient. In addition, similar results were found in the study conducted by Engin et al. [18].

Inflammation is a critical and fundamental process in the development and progression of cancer [10]. In many recent studies, the relationship between the parameters discussed in our study and the survival of various cancer patients has been evaluated. In addition, the inflammatory response to cancer cells is associated with cancer progression. The inflammatory reaction is critical in the regeneration of tumor-damaged tissues and the tumor microenvironment. Inflammatory cells are responsible for cell proliferation, angiogenesis, invasion, migration, and metastasis. Therefore, inflammation plays a crucial role in cancer development and progression [19]. Therefore, many studies have tried to predict the prognosis of colorectal diseases using NLR and PLR biomarkers.

Wu et al. found that increased NLR values adversely affect the prognosis [20]. Therefore, it can be said that it is associated with the growth in the tumor stage. Similarly, in our study, NLR values increased with respect to the tumor stage. Okuyucu et al., Sun et al., and Son et al. concluded in their study that increased NLR rates, similar to our study, could be a predictive biomarker in poor prognosis [15,21,22].

The main limitation is the inconsistency in the cut-off values of the NLR. Cut-off values of 4, 2.9, 2.8, 2.6, and similar were used for NLR in some studies. For example, Shen et al. suggested that 2.8 was the best cut-off value for the NLR to differentiate the prognosis of patients with colorectal cancer, which contrasts with 4.0 in the study by Kaneko et al. In our study, the cut-off value was found to be 3.7 [23-24]. The reason for the different NLR values may be the difference in laboratory measurement values and the number of patients.

Portale et al. investigated 152 patients with rectal cancer who underwent curative resection and reported that PLR is not a valid prognostic factor [16]. However, there have also been studies that suggest otherwise. One study found that decreased preoperative PLR was significantly associated with better mean survival in patients with stage I-III colon cancer. It was concluded that preoperative PLR is an independent prognostic indicator for mean survival in colon cancer patients undergoing curative resection [12]. This was not consistent with this study, as the cancer stage is associated with prognosis. In the study conducted by Hu et al., an increase in PLR values was significant for distinguishing cancer patients from non-cancer patients [25]. However, in our study, no statistically significant relationship was found between the stages of the tumor.

This study inevitably has some limitations due to its retrospective planning and single-center design. However, NLR and PLR values and their relationship with tumor stage, presence of LN metastases, and distant metastases in patients with colon cancer had not been well evaluated previously. In light of that, we aimed to provide clinicians with a new and helpful tool that can be easily accessed and calculated in addition to traditional methods and staging systems when planning individualized treatment for colon cancer.

## Conclusions

Based on our findings, NLR is associated with the tumor stage, and therefore with prognosis. As a result, it has been concluded that increased PLR may not be associated with the colon cancer stage. However, NLR values were found to be significant. NLR values in colon cancer can be an accurate prognostic indicator for predicting clinical stages and optimizing therapeutic strategies. Although the specificity and sensitivity values are currently found to be low, we believe better results can be found with larger studies.

## References

[1] Singh S, Mayengbam SS, Chouhan S, Deshmukh B, Ramteke P, Athavale D, Bhat MK (2020). Role of TNFα and leptin signaling in colon cancer incidence and tumor growth under obese phenotype. Biochim Biophys Acta Mol Basis Dis.

[2] Cokkinides V, Albano J, Samuels A, Ward ME, Thum JM (2005). American Cancer Society: Cancer Facts and Figures. https://www.cancer.org/content/dam/cancer-org/research/cancer-facts-and-statistics/annual-cancer-facts-and-figures/2005/cancer-facts-and-figures-2005.pdf.

[3] O'Connell JB, Maggard MA, Liu JH, Etzioni DA, Livingston EH, Ko CY (2003). Rates of colon and rectal cancers are increasing in young adults. Am Surg.

[4] Rustgi AK (2007). The genetics of hereditary colon cancer. Genes Dev.

[5] Fuchs CS, Giovannucci EL, Colditz GA, Hunter DJ, Speizer FE, Willett WC (1994). A prospective study of family history and the risk of colorectal cancer. N Engl J Med.

[6] Swanson RS, Compton CC, Stewart AK, Bland KI (2003). The prognosis of T3N0 colon cancer is dependent on the number of lymph nodes examined. Ann Surg Oncol.

[7] Cohen AM, Tremiterra S, Candela F, Thaler HT, Sigurdson ER (1991). Prognosis of node-positive colon cancer. Cancer.

[8] Nitsche U, Stögbauer F, Späth C, Haller B, Wilhelm D, Friess H, Bader FG (2016). Right sided colon cancer as a distinct histopathological subtype with reduced prognosis. Dig Surg.

[9] Fichtner-Feigl S, Kesselring R, Strober W (2015). Chronic inflammation and the development of malignancy in the GI tract. Trends Immunol.

[10] Balkwill F, Mantovani A (2001). Inflammation and cancer: back to Virchow?. Lancet.

[11] Diakos CI, Charles KA, McMillan DC, Clarke SJ (2014). Cancer-related inflammation and treatment effectiveness. Lancet Oncol.

[12] Li Z, Xu Z, Huang Y, Zhao R, Cui Y, Zhou Y, Wu X (2019). Prognostic values of preoperative platelet-to-lymphocyte ratio, albumin and hemoglobin in patients with non-metastatic colon cancer. Cancer Manag Res.

[13] Ozmen S, Timur O, Calik I (2017). Neutrophil-lymphocyte ratio (NLR) and platelet-lymphocyte ratio (PLR) may be superior to C-reactive protein (CRP) for predicting the occurrence of differentiated thyroid cancer. Endocr Regul.

[14] Koh CH, Bhoo-Pathy N, Ng KL (2015). Utility of pre-treatment neutrophil-lymphocyte ratio and platelet-lymphocyte ratio as prognostic factors in breast cancer. Br J Cancer.

[15] Okuyucu M, Yücel I (2020). The relationship between peripheral blood count and prognosis in patients with gastric cancer (Article in Turkish). J Istanbul Faculty Med.

[16] Portale G, Cavallin F, Valdegamberi A, Frigo F, Fiscon V (2018). Platelet-to-lymphocyte ratio and neutrophil-to-lymphocyte ratio are not prognostic biomarkers in rectal cancer patients with curative resection. J Gastrointest Surg.

[17] Yıldırım ME, Badem H, Karataş ÖF, Çimentepe E, Ünal D, Özal T (2013). Preoperative value of preoperative neutrophil/lymphocyte ratio in determining the stage of testicular tumors (Article in Turkish). J Turgut Ozal Med Cent.

[18] Kölükçü E, Erdemi̇r F, Kılıç Ş, Fırat F, Eti̇kan İ (2017). The relationship between tumor grade and preoperative neutrophil lymphocyte ratio in patients with testicular tumor (Article in Turkish). Gaziosmanpaşa Tıp Dergisi.

[19] Ikeda M, Furukawa H, Imamura H (2002). Poor prognosis associated with thrombocytosis in patients with gastric cancer. Ann Surg Oncol.

[20] Wu Y, Li C, Zhao J (2016). Neutrophil-to-lymphocyte and platelet-to-lymphocyte ratios predict chemotherapy outcomes and prognosis in patients with colorectal cancer and synchronous liver metastasis. World J Surg Oncol.

[21] Sun ZQ, Han XN, Wang HJ (2014). Prognostic significance of preoperative fibrinogen in patients with colon cancer. World J Gastroenterol.

[22] Son HJ, Park JW, Chang HJ (2013). Preoperative plasma hyperfibrinogenemia is predictive of poor prognosis in patients with nonmetastatic colon cancer. Ann Surg Oncol.

[23] Shen L, Zhang H, Liang L (2014). Baseline neutrophil-lymphocyte ratio (≥2.8) as a prognostic factor for patients with locally advanced rectal cancer undergoing neoadjuvant chemoradiation. Radiat Oncol.

[24] Kaneko M, Nozawa H, Sasaki K (2012). Elevated neutrophil to lymphocyte ratio predicts poor prognosis in advanced colorectal cancer patients receiving oxaliplatin-based chemotherapy. Oncology.

[25] Hu Z, Tan S, Chen S (2020). Diagnostic value of hematological parameters platelet to lymphocyte ratio and hemoglobin to platelet ratio in patients with colon cancer. Clin Chim Acta.

